# Lab-Scale Twin-Screw Micro-Compounders as a New Rubber-Mixing Tool: ‘A Comparison on EPDM/Carbon Black and EPDM/Silica Composites’

**DOI:** 10.3390/polym13244391

**Published:** 2021-12-15

**Authors:** Nazlı Yazıcı, Mehmet Kodal, Güralp Özkoç

**Affiliations:** 1 Department of Chemical Engineering, Kocaeli University, Kocaeli 41001, Turkey; nazliyazici93@gmail.com; 2 Polymer Science and Technology Graduate Program, Kocaeli University, Kocaeli 41001, Turkey; 3 Nanotechnology Research and Application Center, Sabancı University, Istanbul 34956, Turkey; guralp.ozkoc@istinye.edu.tr; 4 Department of Chemistry, Istinye University, Istanbul 34396, Turkey

**Keywords:** lab-scale twin-screw micro-compounder, EPDM, carbon black, silica

## Abstract

The research and development (R&D) in rubber formulation development require reproducible, repeatable, fast, accurate, and efficient sample preparation. The lab-scale formulation development is conventionally carried out using small-scale internal mixers and two-roll mills. However, high torque laboratory twin-screw micro-compounder, which have been serving the plastic industry for more than 30 years, can be used to formulate new rubber compounds for fast and accurate sample preparation that on top can contribute to the economics of R&D. In this study, we investigated the possibility of using lab-scale 15 mL high torque twin-screw micro-compounder as a tool for new rubber compound development. For this purpose, we formulated EPDM/carbon black and EPDM/silica recipes through conventional way using a Banbury mixer followed by a two-roll mill, and through the possible way using a lab-scale 15 mL twin-screw micro-compounder. We crosslinked both systems via hot press at a predefined temperature and time. The rheological and mechanical properties of the compounds were investigated. Moreover, the dispersion of carbon black and silica in the EPDM matrix was judged by DisperGrader and scanning electron microscope (SEM). The conventional way of sample preparation was compared with a possible sample preparation method based on materials’ parameters and ease of operation.

## 1. Introduction

Rubber technology has a history of more than 180 years since the invention of sulphur vulcanization process by Goodyear in the first half of the 19th century. However, different peroxides are also widely used as curing agents to produce rubber products [1,2,3,4,5,6,7]. In addition, crosslinked rubbers can be made using methods such as ionizing radiation. After the vulcanization process, the hardness of the rubber increases, and its viscoelastic, mechanical, and thermal properties are significantly improved [1]. Most industrially produced rubber products are made by sulfur vulcanization and contain many chemicals such as accelerators, process aids, and antioxidants. For example, anti-degradants are used to protect rubber products from degradation and to improve the mechanical properties of rubbers, reinforcing agents such as carbon black and silica are incorporated into rubbers. Changing the loading levels of these chemicals, processable and cost-effective products can be obtained to meet customer needs. To achieve these properties, it is imperative to form the proper rubber formulations and distribute the ingredients homogeneously in rubber compounds. For this purpose, the rubber compounding process is critical in terms of obtaining commercial products.

The global rubber market is expected to grow from USD 27 billion in 2017 to USD 34 billion by 2022, at a compound annual growth rate (CAGR) of 4.6% from 2017 to 2022 [8]. Moreover, academic studies related to rubbers, including patents, proceedings, etc., have significantly increased in the last decade. Therefore, it is believed that rubber science and technology will be one of the essential areas in the coming decade. The research and development (R&D) in rubber formulation development requires reproducible, repeatable, fast, accurate, and efficient sample preparation. As mentioned above, rubber compounding is a conventional process that combines rubbers and various chemicals such as vulcanizing agents, stabilizers, and fillers to produce rubber-based products of the desired properties. Open mills or internal mixers (Banbury) are used to prepare rubber compounds. After that, the resulting compound is shaped on the mills, extruders, or calenders prior to the vulcanization process. One of the most important criteria and complications in rubber compounding is the increasing dispersion of fillers such as carbon black (CB) or silica, and the chemicals such as sulphur, antioxidants, accelerator, etc., in the matrix. To improve the dispersion of additives and fillers in rubber compounds, robust mixers facilitate enough shear and residence time and need to be reproducible, user-friendly, and fast.

Different processing issues such as the mixing process and the type and the size of the mixing equipment should be considered to obtain rubber compounds having desired properties. For instance, Gui and coworkers introduced a new mixing method, latex compounding, for silica/styrene butadiene rubber (SBR) masterbatches. The properties of the compounds obtained by the innovative approach were compared with the dry mixing method. Compared with the dry traditional blending method, the authors observed lower energy consumption and homogenous dispersion of silica during compounding. Moreover, they stated that better dispersion of silica via novel mixing technique resulted in improved mechanical properties. Besides, the excellent dispersion of silica in SBR compounds with this novel technique significantly enhanced the wet skid resistance of the compounds, which could be attributed to the weakens the filler network structure [9]. In a recent study, Noramirah and Azura investigated the effect of combination ultrasonic and ball milling techniques on the dispersion of silica and carbon black for natural rubber (NR) latex compounds. They obtained smaller particle size and finer dispersion of both carbon black and silica in NR compounds prepared by ultrasonic method followed by ball milling method, which positively affected the mechanical properties such as tensile strength and tensile modulus on NR latex films [10].

The lab-scale formulation development is conventionally carried out using small-scale internal mixers and two-roll mills. These conventional compounding methods contain some critical disadvantages, such as being labor and time-intensive (at least two personal) to prepare a sample of one batch, cleaning is a difficult task (two Banbury for black and non-black samples), long turnaround time, etc. In addition, from a safety point of view, two roll miles are dangerous since they have an open mixing area. It needs at least 300–400 cc material; expensive additives are therefore an issue. Besides, both open mills and internal mixers occupy 6 m^2^, mostly separate laboratory space because of CB dusting. However, high torque laboratory twin-screw micro-compounder, which have been serving the plastic industry for more than 30 years, can be used to formulate new rubber compounds for fast and accurate sample preparation that on top can contribute to the economics of R&D. Moreover, micro compounders in rubber compounding at laboratory scale have some other properties such as that one person can operate, easy, less labor-intensive and the compound is ready in 5 min. It needs only small amounts of materials to prepare compounds with expensive materials. More accurate formulations can be prepared. It occupies small lab space as compared to conventional ones. Besides, it is easy to clean and safe to operate.

In this study, for the first time in the literature, we investigated the possibility of using a lab-scale 15 mL high torque twin-screw micro-compounder as a tool for new rubber compound development. For this purpose, we formulated ethylene propylene diene monomer (EPDM)/carbon black (CB) and EPDM/silica recipes through conventional way using a Banbury mixer followed by a two-roll mill, and through the possible way using a lab-scale 15 mL high torque twin-screw micro-compounder. The logic behind the selection of CB and silica is to compare the dispersibility of two different fillers having different compatibilities with EPDM. We crosslinked both systems via hot press at a predefined temperature and time. The rheological and mechanical properties of the compounds were investigated. Moreover, the dispersion of carbon black and silica in the EPDM matrix was judged by DisperGrader and electron microscope. The conventional way of sample preparation was compared with a possible way of sample preparation based on materials parameters and ease of operation as well.

## 2. Materials and Methods

### 2.1. Materials

EPDM (Keltan 9650Q) was purchased from Lanxess. CB was purchased from Elkim. Silica, zinc oxide, stearic acid, 2,2,4-Trimethyl-1,2-Dihydroquinoline (TMQ), Tetramethylthiuram disulfide (TMTD), 2-Mercaptobenzothiazole (MBT) were provided by RubberChem. Paraffinic oil was obtained from Petro Yag, Turkey (Table 1). Rubber compound recipes were given in Table 2.

Three different recipes were prepared. EPDM/CB and EPDM/Silica recipes were prepared to determine the mixing quality in the presence of compatibility (EPDM/CB) and incompatibility (EPDM/Silica) with respect to the processing method.

### 2.2. Processing

#### Compounding by Banbury Mixer Followed by Two-Roll Milling

**Figure 1:**
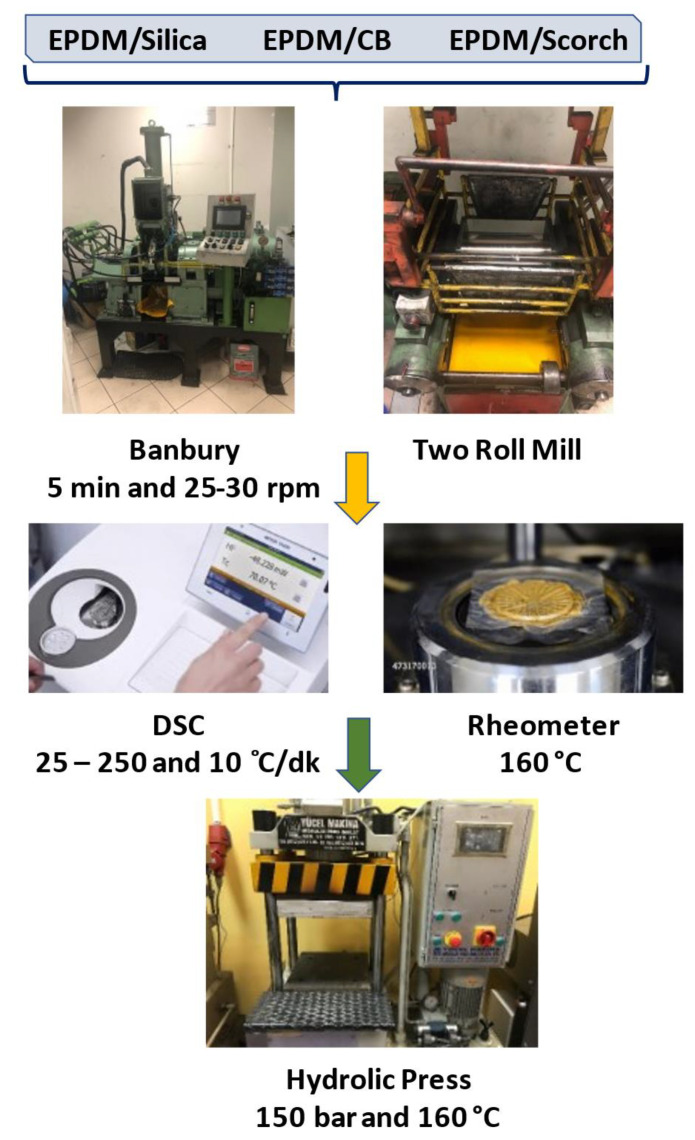
Flow chart of the Banbury compounding.

Compounding EPDM with silica or carbon black was carried out in a 2 L Banbury mixer (Met-Gur model, Turkey) at a 25 rpm of rotor speed and an average temperature of 60 °C. First, EPDM was masticated for 2 min. Silica or carbon black and oil were added to the compound and mixed for 5 min, and then activator and antioxidant were incorporated and were mixed for further 2 min. Finally, accelerator and curing agent were incorporated into the EPDM compounds for 1 min. The complete compounding cycle was approximately 10 min. Rubber compounds were homogenized and shaped by using a laboratory-type high torque two-roll mill having a diameter of 15 cm and length of 30 cm. Milling speed was kept constant at 10 rpm, and the friction ratio was 1:1.1. The temperature was 60 °C, and the distance between the cylinders was varied from 0.5 to 3 mm during mixing. The curing of EPDM composites was carried out in a hot press at 160 °C. The curing temperature and optimum curing time were determined from a differential scanning calorimeter (DSC) and a moving die rheometer (MDR) analysis, respectively (Figure 1). Tensile properties, hardness, thermo-mechanical and thermal analyses were determined from tensile tests, durometer, dynamic mechanical analyzer (DMA), and thermal gravimetric analyzer (TGA), respectively. In addition, the morphological properties of compounds were monitored by scanning electron microscopy (SEM) and DisperGrader.

### 2.3. Compounding Using MC 15 HT

**Figure 2:**
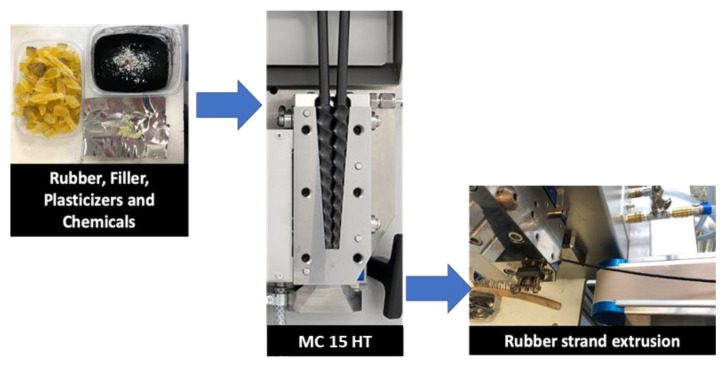
Flow chart of the MC 15 HT compounding.

A lab-scale high torque twin-screw micro-compounder (Xplore Instruments MC 15 HT, The Netherlands) was used to prepare the EPDM-based compounds. The premixed rubber and ingredients, except sulphur and accelerator, were fed to the compounder. The barrel temperature was controlled using water-cooling jackets to keep it at 60 °C. At the end of 2 min, the sulphur and accelerator were added to the compound and mixed for 1 min. The rubber strands were then taken from the die of the compounder by changing the position of the discharge valve. A picture of the compounder can be seen in Figure 2. The curing of EPDM composites was carried out in a hot press at 160 °C. The curing temperature and optimum curing time were determined from differential scanning calorimeter (DSC) and moving die rheometer (MDR) analysis, respectively.

### 2.4. Characterization

The level of dispersion of silica and/or CB in cured EPDM composites was evaluated by scanning electron microscopy (SEM) and DisperGrader analysis. The surface of composites was coated with gold for SEM analysis to prevent the samples from arching. Moreover, to determine the CB and silica dispersion in EPDM and dispersion quality of the cured EPDM samples, DisperGrader analyses were performed according to the ASTM D7723-19.

Thermal properties and curing behaviors of EPDM compounds were investigated by differential scanning calorimeter (DSC, Mettler Toledo DSC-1 Star). The analyses were conducted under N_2_ atmosphere at a heating rate of 10 °C/min between 25 °C and 250 °C.

Important rheological parameters and optimum cure times were obtained from rheometer curves by using a moving die rheometer (MDR, Alpha Technologies) according to ASTM D-5289. Rheometer curves obtained at 160 °C were used to define rheological parameters. Crosslink density (CLD) of the vulcanizates was calculated according to Flory–Rehner Equation using swelling ratios in toluene, the density of rubber matrix, density of toluene, volume fractions of the polymer and solvent, and polymer-solvent interaction parameter, ꭕ.

Tensile properties of cured EPDM composites were determined using an Instron Universal Testing Machine (Model 3345) according to ASTM D412. The crosshead speed was 500 mm/min. The hardness of the samples was measured using Zwick Shore A type durometer according to ASTM D2240.

Dynamic mechanical properties of the EPDM/CB and EPDM/Silica composites were measured using 0.1 db Metravib DMA 50 model device. Measurements were carried out from −100 °C to 40 °C at a frequency of 1 Hz at a heating rate of 5 °C/min.

Thermal stability of the composites was determined using thermal gravimetric analysis (TGA, Mettler Toledo), under N_2_ atmosphere, and at a heating rate of 10 °C/min from ambient temperature to 600 °C.

## 3. Results and Discussions

### 3.1. Scanning Electron Microscopy (SEM) Test Results

**Figure 3:**
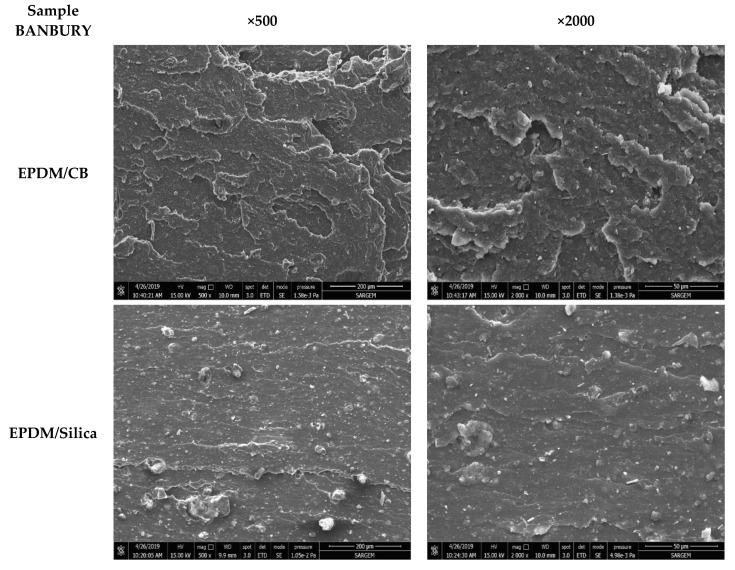
Tensile fractured surface morphologies of the EPDM/CB and EPDM/Silica composites prepared in Banbury (magnification × 500 and × 2000).

**Figure 4:**
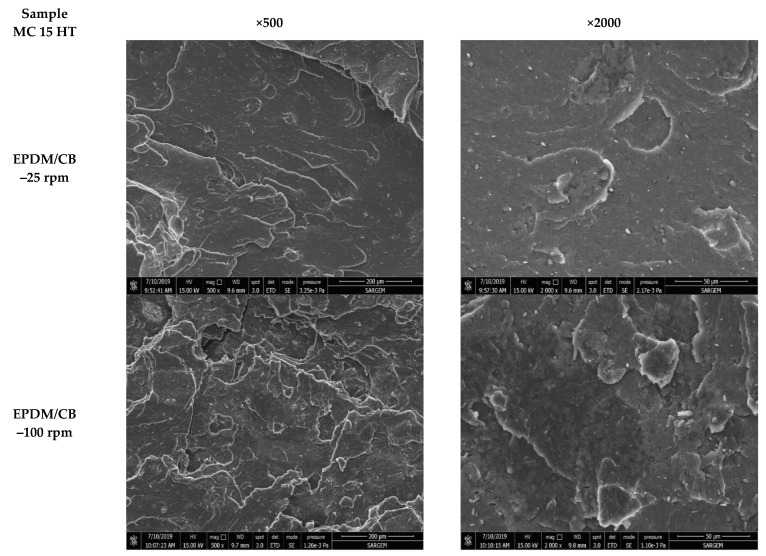
Tensile fractured surface morphologies of the EPDM/CB composites prepared in MC 15 HT (magnification × 500 and × 2000).

**Figure 5:**
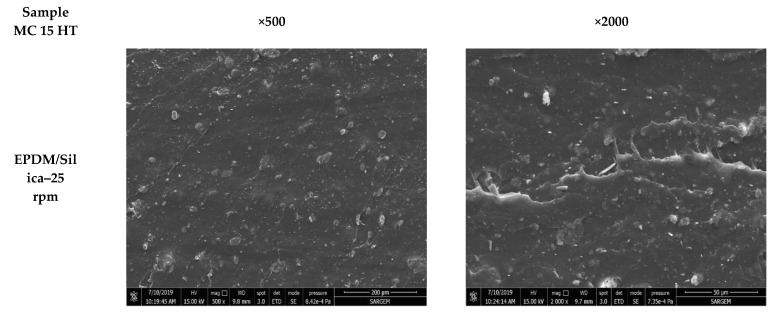

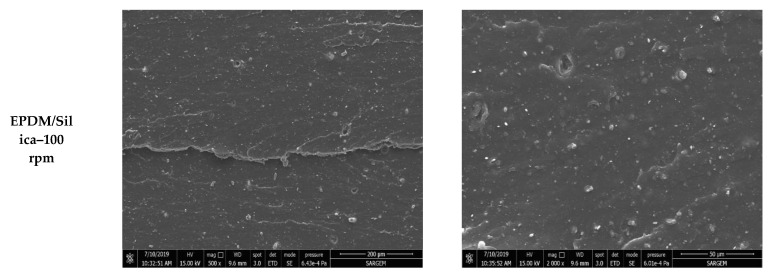
Tensile fractured surface morphologies of the EPDM/Silica composites prepared in MC 15 HT (magnification × 500 and × 2000).

The level of dispersion of CB and silica in EPDM composites prepared in Banbury and MC 15 HT was evaluated by SEM analyses. Figure 3, Figure 4 and Figure 5 shows the SEM micrographs of tensile fractured surfaces of EPDB/CB and EPDM/silica composites prepared in both Banbury and MC 15 HT with different screw speeds.

As can be seen in Figure 3 and Figure 4 that CB was dispersed homogeneously in EPDM matrix independent from the sample preparation method. On the other hand, agglomerated silica particles were obtained compared to CB due to the challenging dispersion of silica in polymers. For EPDM/silica composites prepared in Banbury, silica dispersion was found coarser (Figure 3). The particle size of silica in EPDM ranged from 10 to 110 µm. On the other hand, the preparation of EPDM/silica composites in MC HT 15@25 rpm led to a decrease in the diameter of silica agglomerates (Figure 5). The average diameter of silica was found 20 µm. Moreover, a significant improvement in the dispersion of silica in EPDM composites prepared in MC 15 HT@100 rpm was obtained, as shown in Figure 5.

### 3.2. DisperGrader Test Results

**Figure 6:**
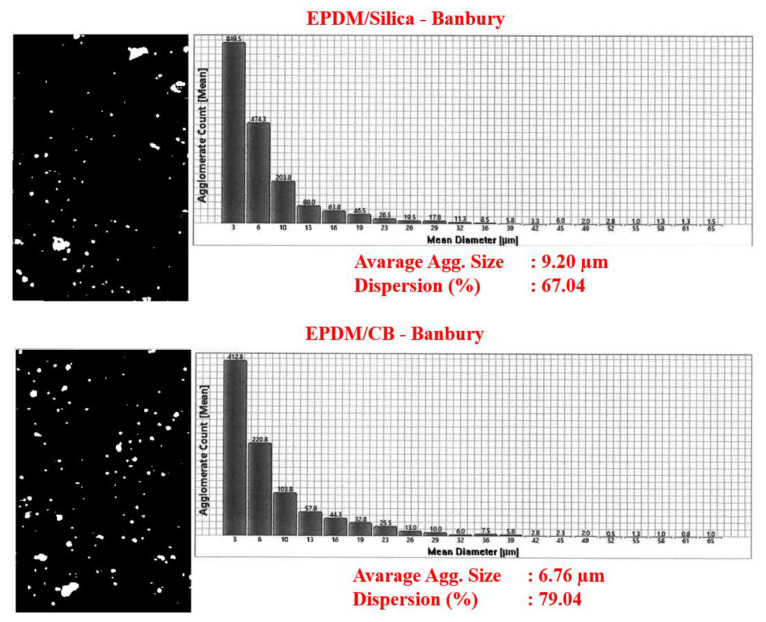
CB and silica dispersion in EPDM composites prepared in Banbury.

**Figure 7:**
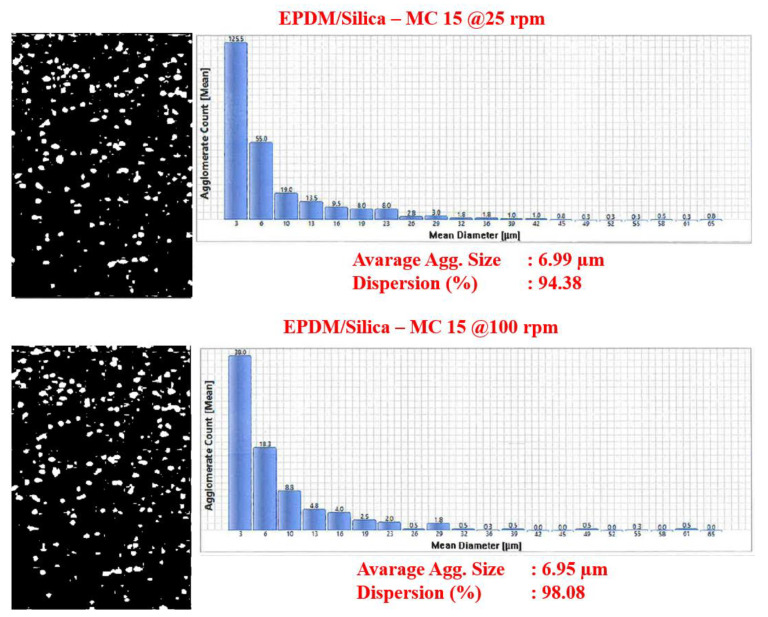
Silica dispersion in EPDM composites prepared in MC 15 HT.

**Figure 8:**
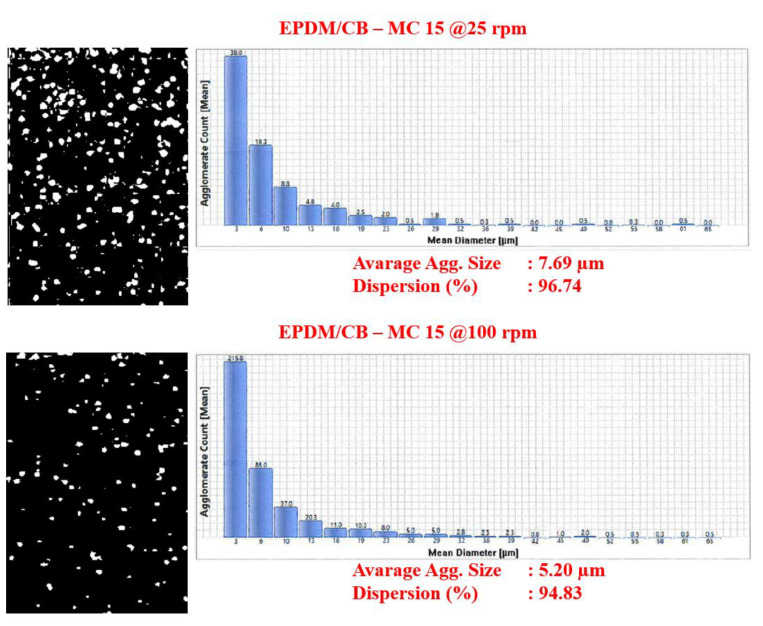
CB dispersion in EPDM composites prepared in MC 15 HT.

To determine the quality of the EPDM compounds and CB and silica dispersion in EPDM, DisperGrader analyses were carried out. Figure 6, Figure 7 and Figure 8 shows the images, average particle size, and percent dispersion obtained from the DisperGrader analysis indicating the dispersion of CB and silica in EPDM.

Generally, the dispersion of CB and silica in a polymer matrix is related to reducing the size of CB and silica in the form of aggregates. It was obtained for EPDM/silica composites that the dispersion of silica in EPDM was much better in the composites prepared with MC 15 HT compared to the Banbury. Moreover, the average particle size of silica decreased significantly from 9.20 µm to 7 µm and, the dispersion quality percentage of silica was much better in the composites prepared in MC 15 HT. Similar test results were also obtained for EPDM/CB composites.

In order to better understand the difference in the level of dispersion of both silica and CB in the EPDM matrix depending on the compounding route, a deeper look into the mixing process in both the Banbury mixer and the co-rotating, intermeshing twin-screw compounder is needed. In a Banbury mixer, the mixing occurs inside a closed chamber with rotating kneading rotors. The Banbury mixer used in this study has tangential rotors that do not intermesh. Dispersive mixing that results in the break-up of the particles into a smaller size is only accomplished in high shear tapering nip regions between rotor tips and the Banbury outer wall, so-called the barrel. Therefore, only a small portion of the melt is always under high shear strength in the Banbury mixer. Distributive mixing that leads to the homogenization of the broken particles (spatial distribution of individual particles) in the matrix occurs by the transfer of material from one rotor to the other where elongational deformation is dominant [11,12,13]. Therefore, in order to achieve good dispersion and distribution of the fillers in a rubber matrix, generally longer mixing times are needed at relatively slow rotor speeds to prevent overheating of the Banbury due to the viscous heat generation. On the other hand, the flow field of an intermeshing co-rotating twin-screw extruder is mostly dominated by simple shear on the radial direction [14] together with the elongational flow on the axial direction. Different than a Banbury mixer, the total amount of the content in an intermeshing twin-screw co-rotating extruder is under continuous shear stresses during processing.

In the current work, as mentioned earlier, the lab-scale intermeshing high torque twin-screw co-rotating compounder (MC 15 HT) yielded better dispersion levels for both silica and CB in comparison to that obtained in Banbury. Based on the information given above that this is possible because the shear intensity of the MC 15 HT is much higher than that of Banbury, as explained above, which could lead to a better dispersion of the fillers in the EPDM matrix. These findings revealed that MC 15 HT could be very useful to prepare rubber compounds for R&D screening purposes.

### 3.3. DSC Results

**Figure 9:**
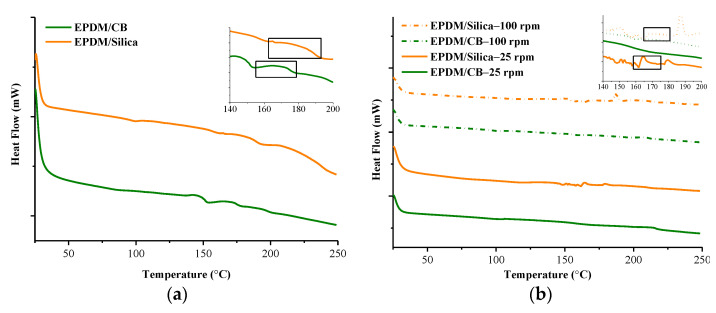
DSC curves of samples (**a**) prepared in Banbury, (**b**) prepared in MC 15 HT.

The DSC curves of EPDM/CB and EPDM/Silica composites prepared in Banbury mixer as shown in Figure 9a and transition values were represented in Table 3. It is seen that the crosslinking exotherm is seen around 155–180 °C for EPDM/Silica and 163–197 °C for EPDM/CB samples. This difference can be due to the difference in the filler content and the filler type that would affect the vulcanization temperature. The vulcanization process during the curing of rubber in a mold is controlled by the diffusion of the reactive species to the reaction sites. If there are physical obstacles, like carbon or silica particles, that could retard the diffusion process, the reaction delays. In the current case, since the content of CB is more than silica, and the dispersion level of CB is better compared to silica, this would cause a possible retardation effect on the vulcanization process due to hindrance of the reaction sites. As a result, the crosslinking temperature shifts to a higher temperature in the case of CB.

The DSC curves of EPDM/CB and EPDM/Silica composites prepared in MC 15 HT were shown in Figure 9b, and transition values were represented in Table 3. As can be seen from Table 3 that curing onset temperature (T_c,o_) of EPDM/CB and EPDM/Silica prepared in MC 15 HT shifted higher temperatures as compared to prepared in Banbury. Moreover, for EPDM samples prepared in MC 15 HT, T_c,o_ also increased as the screw speed increased. These findings can be attributed to the better CB and silica dispersion in the EPDM compound prepared at MC 15 HT at higher rpm as explained earlier. Better dispersion results in higher interfacial surface area between rubber and fillers that plays a role in obstruction on the pathway of diffusion. Hence, the diffusion process retards, in other words, shifts to the higher temperature in DSC analysis.

### 3.4. Rheometer Results

**Figure 10:**
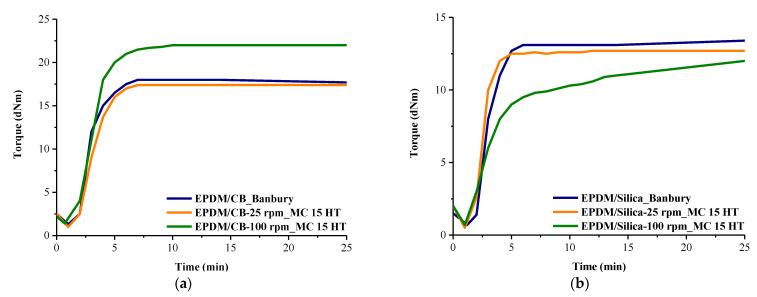
Rheometer curves of the composites (**a**) EPDM/CB prepared in Banbury and MC 15 HT, (**b**) EPDM/Silica, prepared in Banbury and MC 15 HT.

The rheometer results of the EPDM compounds in Figure 10 and corresponding data are represented in Table 4. It is seen that for EPDM/CB compounds, the Banbury and MC 15 HT@25 rpm yielded nearly similar results; however, MC 15 HT@100 rpm exhibited higher MH values in comparison to the samples prepared in Banbury. This can be due to the better dispersion of the CB in EPDM at higher rpm. Moreover, the highest torque difference (MH–ML), which can be related to the crosslink density of the composites, was obtained for EPDM/CB samples prepared in MC 15 HT@100 rpm. This increment in crosslink density can be attributed to the increased interfacial interaction between carbon black and EPDM. Swelling measurements also indicated that EPDM/CB compound prepared in MC 15 HT@100 rpm exhibited the highest crosslinking density (CLD), which was consistent with cure extent values.

For EPDM/Silica, the samples prepared in Banbury and the samples prepared in MC 15 HT@25 rpm showed nearly similar results; however, the samples prepared in MC 15 HT@100 rpm yielded higher t90 with a marching behavior. This is due to the higher interfacial area between EPDM and silica, where silica retards the vulcanization of EPDM. Moreover, the acidic nature of silanol groups of finely dispersed silica particles in EPDM compounds prepared at MC 15 HT@100 rpm significantly affected the curing time and CRI. This situation increased the interaction of silica with the activator, which resulted in higher t90 and lower CRI [15,16]. On the other hand, different from EPDM/CB compounds, finely dispersed silica particles in EPDM compounds prepared in MC 15 HT@100 rpm adversely affected the crosslink density of compounds, as seen in the cure extent values in Table 4. As stated in the literature, silica can react with a cure accelerator via a hydrogen bond. This will accelerate the bond dissociation of N–S [17]. Therefore, the consumption of cure accelerator in the presence of well-dispersed silica particles in EPDM compound led to a decrease in cure extent values of EPDM compound prepared in MC 15 HT@100 rpm. Similar findings were observed from Flory–Rehner’s swelling measurement that the lowest CLD value was found for EPDM/Silica prepared at MC 15 HT@100 rpm.

### 3.5. Mechanical Properties

**Figure 11:**
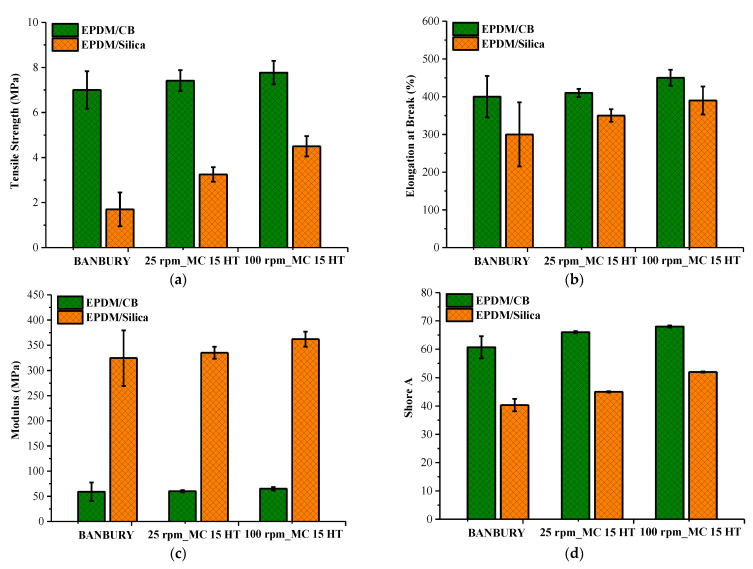
(**a**) Tensile strength values, (**b**) Elongation at break values, (**c**) Young’s modulus, (**d**) Shore A values of samples.

Mechanical properties of EPDM compounds were represented in Figure 11a–d. Tensile strength data reveal that the tensile strength obtained from MC 15 HT samples was higher than that of Banbury (Figure 11a). This was possibly due to the better dispersion of the fillers, both CB and silica. The increasing rpm in MC 15 HT from 25 to 100 resulted in improved tensile strength independently from filler type. As Banbury and MC 15 HT were compared, the difference obtained from silica was more significant than that of CB due to the better dispersion.

Additionally, the scatter of the data obtained from MC 15 HT was lower than that of Banbury due to better homogenization of the sample’s batches, as discussed in the DisperGrader test results. A similar trend was also observed for elongation at break values (Figure 11b). The interfacial interaction between polymer matrix and fillers was a much more pertinent criterion for higher tensile strength and elongation at break values in particle reinforced polymer composites. The interfacial interaction between filler and matrix and the interactions between filler and other combinations of components such as accelerators and activators and level of dispersion of fillers in rubber matrix were significant parameters to obtain improvement in tensile properties of rubber-based composite materials [15,18]. Therefore, higher elongation at break and tensile strength values of both EPDM/CB and EPDM/Silica composites were due to the finer dispersion of fillers in EPDM prepared in MC 15 HT@100 rpm, which enhanced the interfacial interaction between CB-EPDM and Silica–EPDM.

Moreover, it was obtained that the Young modulus of samples prepared in Banbury and MC 15 HT@100 rpm exhibited similar values, which was due to the increased CB and silica dispersion in EPDM at 100 rpm in MC 15 HT (Figure 11c). Besides, Shore A hardness of all samples exhibited similar trends, as can be seen in Figure 11d. The higher stiffness and hardness of both EPDM/CB and EPDM/Silica composites can be attributed to the homogenous dispersion of fillers in EPDM compound prepared in MC 15 HT at higher screw speed, which was due to the restricts the slippage of EPDM chains in the presence of finer dispersed fillers [19].

### 3.6. Dynamic Mechanical Analyses (DMA) Test Results

**Figure 12:**
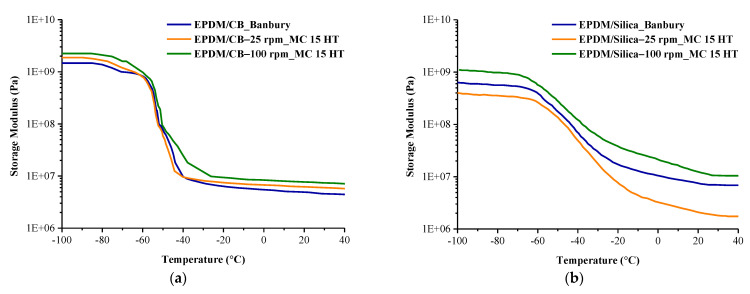
The storage modulus of (**a**) EPDM/CB (**b**) EPDM/Silica composites prepared in Banbury and MC 15 HT.

Viscoelastic properties of the compounds prepared in Banbury and MC 15 HT were determined by DMA analysis. Both the filler–filler and filler–elastomer interactions significantly influence the dynamic mechanical properties of rubber-based composite materials. These interactions depend on the dispersion of fillers in the rubber matrix [20]. As can be seen from Figure 12a,b that the storage modulus of EPDM/CB composites was higher as compared to EPDM/Silica composites prepared in both Banbury and MC 15 HT. This can be attributed to the better interfacial interaction between CB and EPDM than Silica and EPDM.

Moreover, it was obtained higher storage values in EPDM/CB and EPDM/Silica composites prepared in MC 15 HT than that of Banbury, which resulted from the improved dispersion of fillers in MC 15 HT. As discussed in SEM and DisperGrader test results that the particle size of silica decreased in the EPDM matrix as the screw speed of MC 15 HT increased, which resulted in a higher surface area of silica. Therefore, the smaller particle size of silica improved the EPDM–silica interaction that leads to better reinforcing ability [21]. This resulted in higher interfacial interaction between fillers and EPDM. Moreover, the dispersion of silica on the storage modulus of the compounds was obtained much more dominant as a comparison with the effect of crosslink density of vulcanizate.

**Figure 13:**
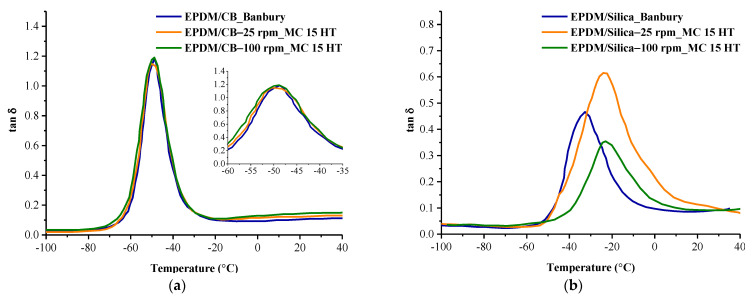
Tan δ of (**a**) EPDM/CB (**b**) EPDM/Silica composites prepared in Banbury and MC 15 HT.

The temperature corresponding to the peak in DMA analysis can be used as the glass transition temperature of EPDM, as shown in Figure 13a,b. As can be seen from Figure 13a,b that the glass transition temperatures of EPDM/Silica composites prepared in Banbury had the lowest because of the bad rubber-filler interaction. In the filled system, filler–polymer and filler–filler interactions lead to an effective immobilization of the elastomer segments [22]. The glass transition temperatures of EPDM/Silica composites prepared in MC 15 HT were all higher than those prepared in Banbury because of better rubber-filler interaction resulting from the homogeneous dispersion of fillers in EPDM. Moreover, the glass transition temperature of EPDM/Silica prepared in MC 15 HT shifted to higher temperatures, which resulted from restricting the mobility of the EPDM chains in the presence of homogeneously dispersed silica particles [23]. In addition, it was observed that the amplitude of tan δ curves of EPDM/Silica prepared in MC 15 HT@100 rpm was the lowest compared to other ones Figure 13b. This finding was also another indication of improved interfacial interaction between silica and EPDM, which increased the storage modulus of EPDM [24,25,26]. On the other hand, different from the EPDM/Silica composites, EPDM/CB composites independent from the mixing device exhibited similar glass transition temperatures, as shown in Figure 13a. Because the dispersion quality of carbon black was found nearly the same in EPDM prepared both Banbury and MC 15 HT as shown in SEM pictures (Figure 3 and Figure 4).

### 3.7. TGA Results

**Figure 14:**
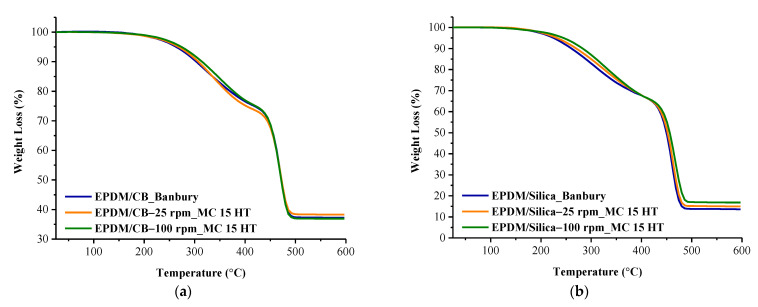
TGA curves of (**a**) EPDM/CB (**b**) EPDM/Silica composites prepared in Banbury and MC 15 HT.

Figure 14a,b shows the TGA curves of the EPDM/CB and EPDM/Silica composites prepared in Banbury and MC 15 HT. A summary of the TGA results, such as T_d5_ (decomposition temperature where 5% weight loss occurred), is shown in Table 5.

Two mass loss steps were obtained for all composites. As can be seen from Table 5, the thermal decomposition temperatures of EPDM/Silica composites prepared in MC 15 HT@25 rpm exhibited higher values than those prepared in Banbury. Moreover, better silica dispersion in EPDM prepared in MC 15 HT@100 rpm resulted in an increasing effect on the decomposition temperature of EPDM. Furthermore, the EPDM/silica sample prepared in MC 15 HT@100 rpm presents a higher char yield, which shows that its decomposition rate is lower as compared to other ones. Similar results were also obtained for EPDM/CB composites. All these findings were due to the increased pathway for volatile decomposition products in the presence of well-dispersed CB and silica in the EPDM matrix prepared in MC 15 HT. These results are also consistent with the literature that well-dispersed fillers in vulcanizates can improve the thermal stability of the polymer composites [15,27,28,29].

## 4. Conclusions

Laboratory-scale mixing devices, which serve as cost and time-efficient research facilities, provide possibilities for processing a few grams of material. They have the capability of continuous or batch processing with the same thermal environment as a conventional extruder. Micro-devices can be used as fast screening tools in the field of polymer and rubber-based nanocomposite development. In this study, EPDM/CB and EPDM/silica composites were prepared in both a conventional rubber-mixing device and an MC 15 HT micro compounder. Moreover, different screw speeds were used to prepare EPDM compounds at MC 15 HT to evaluate the effect of rpm on the dispersion of CB and silica in the EPDM matrix.

SEM analyses showed that significant improvement in the silica dispersion in EPDM composites prepared in MC 15 HT@100 rpm was obtained. Besides, the dispersion quality percentage of both silica and CB was found to be much better in the composites prepared in MC 15 HT. DSC analyses revealed that the curing onset temperature of EPDM prepared in MC 15 HT shifted to higher temperatures compared to prepared in Banbury, which was due to the better dispersion of fillers prepared in MC 15 HT. Mechanical test results revealed that the tensile strength obtained from MC 15 HT samples was higher than that of samples prepared in Banbury. DMA analysis showed that increased silica dispersion in EPDM resulted in higher storage modulus and higher glass transition temperature values. Better silica dispersion in EPDM prepared in MC 15 HT@100 rpm resulted in an increasing effect on the decomposition temperature of EPDM.

As a general conclusion, it can be concluded that compared to the traditional rubber mixing method, the lab-scale high torque twin-screw micro-compounders as a new rubber-mixing tool can be convenient and efficient to prepare both carbon black- and silica-based rubber composites containing finely dispersed silica and CB. The MC 15 HT micro compounder has extensive application potential in the fields of rubber reinforcement, high-performance automotive tires, light rubber products, and other advanced materials, shortly said, a time-efficient R&D screening tool.

## Figures and Tables

**Table 1:** Information on materials used in this study.

Materials	Suppliers
EPDM (9650 Q)	Lanxess/Keltan
Silica	RubberChem
CB	Elkim/N330
Paraffinic Oil	Petro Yag/Octopus N480
Zinc Oxide	RubberChem
Stearic Acid	RubberChem
TMQ	RubberChem
TMTD	RubberChem
MBT	RubberChem

**Table 2:** Rubber compound recipes (phr).

EPDM/Silica		EPDM/CB	
EPDM	100	EPDM	100
Silica	25	CB	100
Oil	60	Oil	60
ZnO	5	ZnO	5
Stearic Acid	2	Stearic Acid	2
TMQ	1	TMQ	1
TMTD	1.5	TMTD	1.5
MBT	0.5	MBT	0.5
Sulphur	2.5	Sulphur	2.5

**Table 3:** DSC results of EPDM/CB and EPDM/Silica composites prepared in Banbury mixer or MC 15 HT.

	T_c,o_ (°C) *	T_c,p_ (°C) *	T_c,e_ (°C) *
BANBURY			
EPDM/Silica	155.7	168.1	180.1
EPDM/CB	162.9	179.5	197.0
MC 15 HT			
EPDM/Silica-25 rpm	163.9	164.3	180.9
EPDM/Silica-100 rpm	185.1	187.4	192.6
EPDM/CB-25 rpm	168.0	213.1	230.3
EPDM/CB-100 rpm	170.0	208.8	218.0

* T_c,o_, T_c,p_, T_c,e_ are standing for curing onset temperature, curing peak temperature, and curing end-set temperature, respectively.

**Table 4:** Data obtained from MDR for the samples prepared in Banbury and MC 15 HT.

	Materials	ML (dNm)	MH (dNm)	t_90_ (min)	Cure Extent (dNm)	CRI	CLD (mol/m^3^)
BANBURY	EPDM/Silica	0.79 ± 0.02	13.40 ± 0.15	4.90 ± 0.18	12.61 ± 0.13	37.04 ± 0.33	113.0 ± 14
	EPDM/CB	1.00 ± 0.11	17.40 ± 0.61	4.90 ± 0.43	16.40 ± 0.52	41.67 ± 3.18	215.0 ± 11
MC 15 HT	EPDM/Silica-25 rpm	0.50 ± 0.04	12.70 ± 0.31	3.80 ± 0.83	12.20 ± 0.27	52.63 ± 0.08	112.6 ± 13
	EPDM/Silica-100 rpm	0.70 ± 0.01	12.20 ± 0.03	13.00 ± 0.66	11.50 ± 0.04	8.93 ± 0.01	105.1 ± 10
	EPDM/CB-25 rpm	2.01 ± 0.07	18.40 ± 0.56	5.00 ± 0.57	16.39 ± 0.62	34.48 ± 0.49	217.1 ± 12
	EPDM/CB-100 rpm	1.40 ± 0.12	22.10 ± 0.95	5.00 ± 0.50	20.70 ± 0.84	33.33 ± 1.45	223.4 ± 12

**Table 5:** TGA results of the composites.

		T_d5_	T_d10_	T_max_	Char Yield
		(°C)	(°C)	(°C)	(%)
BANBURY	EPDM/Silica	225.5	260.7	461.2	13.7
	EPDM/CB	262.8	302.2	470.8	37.2
MC 15 HT	EPDM/Silica 25 rpm	230.5	270.4	463.7	15.0
	EPDM/Silica 100 rpm	245.2	283.0	466.7	16.9
	EPDM/CB 25 rpm	268.2	306.9	470.9	38.3
	EPDM/CB 100 rpm	274.1	314.4	469.6	36.9

## Data Availability

The data presented in this study are available upon request from the corresponding author.
